# Case of Emphysema, in Which the Stethoscope Afforded a Valuable Diagnosis

**Published:** 1827-01

**Authors:** James Sym

**Affiliations:** Surgeon, Kilmarnock, Ayrshire.


### EMPHYSEMA.

*Case of Emphysema, in which the Stethoscope afforded a valuable Diagnosis.* By JAMES SYM, Surgeon, Kilmarnock, Ayrshire.

IN Mr. BELL's case of Emphysema, published in the Number of this Journal for September, the general symptoms were sufficiently distinct to prove that air existed in the pleural sac. In the following complicated case, the evidence obtained from the general symptoms alone would have been exceedingly perplexing, had I not employed the stethoscope, from which a clear and certain diagnosis was obtained.

On the 26th April, 1826, a tradesman, fifty years of age, and subject during the last twelve years to asthma, originating in an injury of the chest, fell down a stair upon his left side. The accident happened at four o'clock P.M.; and, although the pain in his side was so acute that he could not breathe without moaning or even crying out, this did not excite much alarm, because he was in a state of intoxication. Accordingly, I was not called till about ten o'clock, when I found that the pain was situated at the angle of the ninth rib, that emphysema had occurred, and that he had expectorated blood. I could neither detect crepitation of the bone nor discontinuity of the surface by the touch, but the stethoscope communicated to the ear a distinct jerk, at the moment each expiration was completed, and when the chest began to expand for a new inspiration. The emphysema could be traced over the back, into the left axilla, across both breasts, (which sounded, when struck, like inflated bladders,) and up the front of the neck, (which presented a broad flattened aspect.) Upon applying either the ear or the stethoscope to the surface of the emphysematous swelling, the air was heard moving with a crackling noise through the cellular membrane, during the motions of the chest; and, when the intervening air was pressed aside, and the stethoscope kept steadily fixed over the ribs, the respiratory sound transmitted from the lungs was loud and vocal. There was no pectoriloquy. He coughed a little, and his asthma was very distressing, although he said it had often been as bad. Pulse 100, strong, hard, and full; tongue dry and brown in the middle; urgent thirst; face flushed.

Compress and bandage. Venesection to ℥ xxx. Draught of Laudanum and Antimonial Wine. Epsom Salts in the morning.

27th.—In the morning, I found his pulse eighty-eight and soft, and he breathed more easily; but he had passed a restless night, and the cough had been very annoying. Emphysema extended over the face and arms. After pressing aside the swelling from the sternum, the sound emitted by percussion was found to be natural. Respiratory sound of the lungs still clear, both at the seat of the injury and elsewhere. Respirations twenty-four in the minute; expirations laborious, attended with asthmatic wheezing, and occupying double the time of the inspirations.

In the evening, he complained much of pain in his side, and his pulse rose to 100.

Venesection to ℥ xij.

28th.—Expectorates a little mucus, and is less uneasy. Pulse 112, respirations twenty-eight. Seems still to labour under a considerable degree of asthma; and, before expectorating, the bronchial tubes are so much distended by mucus, that they emit only a rattle, whilst the sound emitted by the other parts of the chest is clear, and vocal in some places, in others sibilous.

29th.—I was sent for at two o'clock in the morning. His friends had crowded into his room, in expectation of his death. His mind and expression of countenance were extremely anxious. Complained of an overwhelming sense of oppression in the chest, difficulty of breathing, and a threatening of syncope. Respirations hurried, wheezing, and rattling, with mucus, of which he had several times expectorated a little. Could not bear the smallest deviation from the erect posture of his chest. Pulse rapid; face flushed; skin hot. The distress he seemed to suffer from defective respiration was so agonising, that I apprehended internal emphysema; and it was only by means of auscultation that I could ascertain whether the aggravation of symptoms ought to be partly attributed to that cause, or entirely to the accession of an asthmatic paroxysm, rendered unsually severe by the injury of his lungs and the alarmed state of his mind. Upon applying the stethoscope, I perceived distinctly the respiratory sound down to the very seat of the injury, where it was loudly sibilous. I therefore bled him to ten ounces; tightened his bandage, which he had undone; scarified the skin below the left clavicle, in order to relieve the bronchial tubes and trachea from confinement; dismissed all his visitors except a single attendant; and allayed the excitement of his mind by assuring him that he was not threatened with suffocation, as he himself apprehended, and that he only laboured under one of his fits of asthma. In this opinion I was speedily confirmed by the relief he derived from a copious expectoration of mucus; after which the cure proceeded without any other untoward occurrence, and in the course of three weeks the emphysema had disappeared.

*Remarks.*—The value of the diagnosis obtained from the stethoscope in this case will, I think, be admitted, when we reflect that the symptoms which presented themselves on the morning of the 29th closely resembled those occasioned by effusion of air into the pleural sac; and that, when this occurs to such an extent as to endanger the patient's life, we are directed by the best surgical authorities to puncture the pleura between two of the ribs. Had this operation been performed, it is probable that an additional wound would have been inflicted upon the lung; because the existence of the respiratory sound close upon the fracture of the rib, combined with the previous history of the case, affords ground for suspecting that the lung adhered extensively to the parietes of the thorax.

